# Necessity and Contingency in Developmental Genetic Screens: EGF, Wnt, and Semaphorin Pathways in Vulval Induction of the Nematode *Oscheius tipulae*

**DOI:** 10.1534/genetics.119.301970

**Published:** 2019-01-30

**Authors:** Amhed M. Vargas-Velazquez, Fabrice Besnard, Marie-Anne Félix

**Affiliations:** Institut de Biologie de l’Ecole Normale Supérieure, Ecole Normale Supérieure, Centre National de la Recherche Scientifique, Institut National de la Santé et de la Recherche Médicale, PSL Research University, 75005 Paris, France

**Keywords:** *C. elegans*, *O. tipulae*, *lin-3/EGF*, Wnt, plexin, semaphorin, evolution of development, genetic screens

## Abstract

Genetic screens in the nematode *Caenorhabditis elegan*s identified the EGF/Ras and Notch pathways as central for vulval precursor cell fate patterning. Schematically, the anchor cell secretes EGF, inducing the P6.p cell to a primary (1°) vulval fate; P6.p in turn induces its neighbors to a secondary (2°) fate through Delta-Notch signaling and represses Ras signaling. In the nematode *Oscheius tipulae*, the anchor cell successively induces 2° then 1° vulval fates. Here, we report on the molecular identification of mutations affecting vulval induction in *O. tipulae*. A single Induction Vulvaless mutation was found, which we identify as a *cis*-regulatory deletion in a tissue-specific enhancer of the *O. tipulae lin-3* homolog, confirmed by clustered regularly interspaced short palindromic repeats/Cas9 mutation. In contrast to this predictable Vulvaless mutation, mutations resulting in an excess of 2° fates unexpectedly correspond to the plexin/semaphorin pathway. Hyperinduction of P4.p and P8.p in these mutants likely results from mispositioning of these cells due to a lack of contact inhibition. The third signaling pathway found by forward genetics in *O. tipulae* is the Wnt pathway; a decrease in Wnt pathway activity results in loss of vulval precursor competence and induction, and 1° fate miscentering on P5.p. Our results suggest that the EGF and Wnt pathways have qualitatively similar activities in vulval induction in *C. elegans* and *O. tipulae*, albeit with quantitative differences in the effects of mutation. Thus, the derived induction process in *C. elegans* with an early induction of the 1° fate appeared during evolution, after the recruitment of the EGF pathway for vulval induction.

## Abstract (precis)

Genetic screens in the nematode *Caenorhabditis elegans* have identified EGF and Notch pathways as key for vulval precursor cell fate patterning. Here, Vargas-Velazquez, Besnard, and Félix report on the molecular identification of...

HOW multicellular organisms arise from single cells is a question that has intrigued scientists over the ages. In the 1960s, Sydney Brenner selected *Caenorhabditis elegans* as a new model organism to study animal development using genetics (Brenner 1974). Vulva precursor cell (VPC) fate patterning rapidly became one of the most studied developmental processes in *C. elegans*, due to the easy isolation of mutants with a defective vulva (Sternberg 2005).

The *C. elegans* vulva is an epidermal specialization that develops from a row of six VPCs in the ventral epidermis, called P3.p–P8.p from anterior to posterior. In most animals, the central vulval fate, or primary (1°) fate, is adopted by P6.p, while the outer vulval fate, or secondary (2°) fate, is adopted by its neighbors P5.p and P7.p (Sulston and Horvitz 1977; Sternberg 2005). Finally, P3.p, P4.p, and P8.p are able to replace the central cells (for example if they are destroyed with a laser), but normally adopt an epidermal fate with one division and fusion of the daughters to the large epidermal syncytium hyp7 (Sulston and White 1980). Laser ablation of the anchor cell in the gonad primordium results in all precursor cells adopting a tertiary (3°) fate, showing that the vulval fates are induced by the anchor cell (Kimble 1981).

Upon random chemical mutagenesis, some recurrent phenotypes were isolated with pronounced defects in vulval development, such as the Vul (Vulvaless) and Muv (Multivulva) phenotypes (Horvitz and Sulston 1980; Ferguson and Horvitz 1985). The Vulvaless mutants can be easily seen in the dissecting microscope by the internal hatching of the progeny in their mother (bag of worms). The Vulvaless mutants can be further classified in two classes: (i) those that mimick an anchor cell ablation (cells adopting a 3° fate) or Induction Vulvaless, and (ii) those that prevent the development of competent VPCs or Generation Vulvaless (Ferguson *et al.* 1987). The Multivulva mutants are recognized by the additional protrusions on the ventral cuticle (pseudovulvae).

The *C. elegans* Induction Vulvaless and Multivulva mutants allowed the identification of the EGF/Ras/MAP kinase pathway, the former class corresponding to a loss of activity in the pathway and the latter to a gain of activity (Sternberg 2005). In addition, mutations at the *lin-12* locus specifically affect 2° fates: reduction-of-function *lin-12* alleles transform 2° fates to 1° or 3°, while gain-of-function alleles transformed 1° and 3° fates to the 2° fate (Greenwald *et al.* 1983). *lin-12* was shown to encode a Notch receptor, receiving Delta signals mostly produced by P6.p. Studies of the interplay between the EGF and Delta/Notch pathways in the patterning vulval cell fates have established this system as a textbook example of intercellular signaling and organogenesis (Sternberg and Han 1998).

Since the 1990s, studies of vulval development in different *Caenorhabditis* species and other nematode genera have made vulval development an emblematic example of developmental system drift (True and Haag 2001): while the vulval cell fate pattern remains overall invariant (2°1°2° for P5.p, P6.p, and P7.p), evolution occurs in the manner in which it forms. First, the size of the competence group varies (Sternberg and Horvitz 1982; Sommer and Sternberg 1996; Félix *et al.* 2000a; Delattre and Félix 2001; Pénigault and Félix 2011a). Second, vulval cell fate patterning does not always require the anchor cell (Sommer and Sternberg 1996; Félix *et al.* 2000a). Third, when it requires the gonad, ablating the anchor cell at intermediate timepoints has widely different effects depending on the species (Sommer and Sternberg 1994; Félix and Sternberg 1997; Sommer 2005; Kiontke *et al.* 2007; Félix 2012; Félix and Barkoulas 2012). In particular, in many genera of rhabditids and diplogastrids (Félix and Sternberg 1997, 1998; Sigrist and Sommer 1999; Félix *et al.* 2000a; Félix 2007; Kiontke *et al.* 2007), gonad ablation at an intermediate timepoint results in P(5–7).p adopting a 2° fate (*vs.* a 3° fate for the outer cells), with no apparent differences among these three cells. This contrasts with anchor cell ablation results in *C. elegans*, where no such intermediate state exists and P6.p adopts a 1° fate earlier, thereby activating lateral induction and inhibition (Félix 2007) (Figure 1). The mode of induction where an intermediate fate is found for all cells has been called a two-step induction (Félix and Sternberg 1997). In this case, the second step of induction of the 1° fate occurs after one division round on P6.p daughters. Even though two steps can be distinguished (induction of P6.p to a 2° fate then after P6.p division to a 1° fate), signaling from the gonad may be continuous (Félix and Sternberg 1997; Sigrist and Sommer 1999; Kiontke *et al.* 2007).

**Figure 1:**
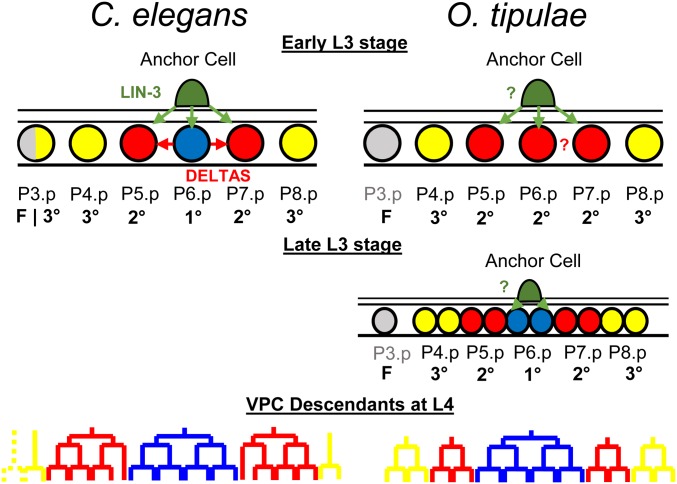
Vulval cell fate patterning in *C. elegans* and *O. tipulae*. In the third larval stage (L3) of *C. elegans*, a cell from the somatic gonad known as the anchor cell (AC) produces an EGF-like inductive signal (LIN-3, green arrows) that activates the Ras pathway in the central vulval precursor cells (VPCs). High Ras signaling promotes the primary (1°) fate (blue circle) in P6.p, which, in turn, produces Deltas (red arrows) that induce a secondary (2°) fate (red circle), and represses the 1° fate in P5.p and P7.p. Both fates prevent the formation of nonspecialized epidermis [tertiary (3°) fate, yellow circles]. Only VPCs with 1° or 2° fates will give rise to the cells that will form the vulva (bottom). P3.p is not competent to acquire a vulval cell fate (gray) in *O. tipulae*. Unlike in *C. elegans*, the AC of *O. tipulae* has been shown to be required to induce the 1° fate in P6.p descendants after VPC division. While a similar vulval cell fate pattern is conserved between the two nematodes, the cell division patterns of the 2° and 3° fates are different.

Among species with a two-step induction (Figure 1), *Oscheius tipulae* is a rhabditid nematode found in the same habitat as *C. elegans* (Félix and Duveau 2012), which can be cultured in the same laboratory conditions (Félix *et al.* 2000b). In this species, the anchor cell first induces P(5–7).p to a 2° fate (first induction) then induces P6.p daughters to a 1° fate (second induction) (Figure 1). A genetic screen was conducted 20 years ago to isolate vulva development mutants in *O. tipulae* (Dichtel *et al.* 2001; Louvet-Vallée *et al.* 2003; Dichtel-Danjoy and Félix 2004a,b). This genetic screen led to a distinct spectrum of vulval cell fate and lineage phenotypes compared to those found in *C. elegans*. This result suggested a different sensitivity of the developmental system to mutation in the two species and therefore a different evolutionary potential, as the phenotypic response to mutation may bias phenotypic evolution (Dichtel-Danjoy and Félix 2004a). The distinct spectrum of mutant phenotypes also reflected the difference in vulval development between *O. tipulae* and *C. elegans* (Dichtel-Danjoy and Félix 2004a). We further identified a null mutant in the Hox gene *lin-39* with the same phenotype as in *C. elegans*, namely a loss of competence of the VPCs (Louvet-Vallée *et al.* 2003).

A draft of the *O. tipulae* genome has recently been published, along with a strategy to map the genomic location of loci whose mutation produces a visible phenotype (Besnard *et al.* 2017). As a proof-of-principle for the mutant identification technique, we described alleles of the *Oti-mig-13* locus with an unexpected vulva phenotype (Besnard *et al.* 2017). Here, we take advantage of the mapping approach to molecularly identify the collection of *O. tipulae* mutations affecting vulval cell fate patterning. We had found a single Induction Vulvaless locus with a single allele and this turned out to be a *cis*-regulatory deletion in a tissue-specific enhancer of the *O. tipulae lin-3* homolog, which we confirmed by targeted clustered regularly interspaced short palindromic repeat (CRISPR) mutation of the element. We then identified mutations in Wnt pathway components (*mom-5/frizzled*, *mig-14/wingless*, and *egl-20/Wnt*) affecting fates of the *O. tipulae* VPCs, and discuss similarities and differences with *C. elegans* and *Pristionchus pacificus*, another nematode species where similar screens have been conducted (Sommer 2006). Finally, the last class of vulval cell fate mutations caused an excess of 2°-fated cells. Unexpectedly, these mutations corresponded to lesions in *Oti-plx-1* and *Oti-smp-1*, encoding plexin and semaphorin, a cell signaling system that was not found in *C. elegans* vulva mutagenesis screens.

## Materials and Methods

### Nematode culture

*C. elegans* and *O. tipulae* were handled according to the usual procedures, on standard NGM plates with *Escherichia coli* strain OP50 as a food source (Brenner 1974; Félix *et al.* 2000b). *C. elegans* and *O. tipulae* strains were maintained, respectively, at 20 and 23°, unless otherwise indicated. N2 was used as a reference strain for *C. elegans* and CEW1, a wild isolate from Brazil, as a reference strain for *O. tipulae*. The *O. tipulae* mutants were all described previously (Dichtel *et al.* 2001; Louvet-Vallée *et al.* 2003; Dichtel-Danjoy and Félix 2004a,b). Except for the Vulvaless *Oti-iov-1(mf86)* mutant, the mutations were all backcrossed one to five times. A list of strains used in this study is presented in Supplemental Material, Table S1.

### Mapping-by-sequencing and identification of molecular lesions

The mapping-by-sequencing strategy using the partial genome assembly of *O. tipulae* includes genetic mapping and whole-genome sequencing in a single step, and has been comprehensively described before (Besnard *et al.* 2017). In brief, each mutant *O. tipulae* line previously obtained in the CEW1 genetic background was crossed to males of the molecularly divergent wild isolate JU170. In the case of the fully Vulvaless *iov-1(mf86)* mutant, males of strain JU432 of genotype *iov-1(mf86)*; *him(sy527)* were crossed to JU170 hermaphrodites. In all cases, individual F2 progeny with a recessive mutant phenotype were isolated and the mutant phenotype verified on the F3 brood. The pooled DNA of the progeny of mutant F2s was extracted using the Puregene Core Kit A (QIAGEN, Valencia, CA) and whole-genome sequenced at the Beijing Genomics Institute facilities. Pools from 37 to 152 individual F2s were used, depending on the ease of scoring of the mutant phenotype.

Sequencing reads from each mutant pool were mapped to the CEW1 genome using bwa (Li and Durbin 2009) and the resulting alignment converted to bam format using samtools (Li *et al.* 2009). Each mapping was further processed with the GATK suite (Van der Auwera *et al.* 2013) and allelic variants were called using HaplotypeCaller on a restricted list of JU170 sites for faster computation. Scaffolds having a mean JU170 allele frequency of < 10% were selected as candidates for possible linkage with a causative locus and processed for homozygous variant calling in an unrestrictive manner. JU170 variants were filtered out from the candidate scaffolds and the remaining variants were analyzed for any functional impact on the *O. tipulae* gene annotations (CEW1_nOt2) using snpEff (Cingolani *et al.* 2012). Scripts used for this processing pipeline can be found at: https://github.com/fabfabBesnard/Andalusian_Mapping. The candidate scaffolds were also analyzed using Pindel (Ye *et al.* 2009) to identify large deletions or insertions, which were confirmed later by visual inspection with the Tablet software (Milne *et al.* 2013).

The genetic loci with names such as *cov-3* and *cov-4* were defined by genetic complementation tests (Dichtel *et al.* 2001; Louvet-Vallée *et al.* 2003; Dichtel-Danjoy and Félix 2004a,b). When the locus was defined by more than one mutant allele, as described in Besnard *et al.* (2017) for *cov-3*, we subjected two alleles to the genetic mapping-by-sequencing approach and determined the intersection between the resulting lists of candidate genes with lesions in the genetic interval, thus pointing to a single common gene. As indicated in the legend to Table 1, for *dov-4(sy451)* and *iov-3(mf78)*, a candidate gene found by the genetic mapping-by-sequencing approach using one or two other allele(s) was tested by PCR for the presence of a lesion and in all cases we found a lesion in the same gene. Finding two noncomplementing alleles with a lesion in the same gene makes it highly likely that we have identified the correct causal locus. The mutations identified by the mapping-by-sequencing approach were also verified by Sanger sequencing of a PCR product. A list of primers used for sequencing can be found in Table S2.

**Table 1:** *O.tipulae* vulval loci identified by the mapping-by-sequencing approach

Locus*^a^*	Allele (method)	Phenotypes	Mutagen	Position*^b^*	Mutation	*Oti* gene	*Cel* homolog	Type of lesion	Reported before in
*cov-4*	*sy465*	Competence loss, P5.p centering	EMS	**10**:202,548	G/A	g06014	*mom-5/frizzled*	Premature stop	Louvet-Vallée *et al.* (2003)
*cov-4*	*sy493*	Competence loss, P5.p centering	EMS	**10**:201,110	C/T	g06014	*mom-5/frizzled*	Splice acceptor	Louvet-Vallée *et al.* (2003)
*cov-5*	*mf34*	Competence loss, P5.p centering	EMS	**3**:189,504	T/C	g01986	*mig-14/Wntless*	Missense variant	Louvet-Vallée *et al.* (2003)
*dov-4*	*sy464*	P4.p/P8.p do not divide, some P5.p centering	EMS	**4**:882,344	G/A	g02936	*egl-20/Wnt*	Premature stop	Dichtel *et al.* (2001)
*dov-4*	*sy451*	P4.p/P8.p do not divide, some P5.p centering	EMS	**4**:882,874	T/C	g02936	*egl-20/Wnt*	Missense variant	Dichtel *et al.* (2001)
*iov-1*	*mf86*	Hypoinduction	TMP-UV	**39**:154,942–155,133	191-bp deletion	g12432	*lin-3*	*Cis*-regulatory deletion	Dichtel-Danjoy and Félix (2004b)
*iov-2*	*mf76*	Hyperinduction 2°	TMP-UV	**10**:74,243–74,877	634-bp deletion	g05993	*smp-1/semaphorin*	Putative null	Dichtel-Danjoy and Félix (2004b)
*iov-3*	*sy447*	Hyperinduction 2°	EMS	**86**:24,647	A/T	g14741	*plx-1/plexin*	Missense variant	Dichtel-Danjoy and Félix (2004b)
*iov-3*	*mf52*	Hyperinduction 2°	EMS	**86**:24,066	A/T	g14741	*plx-1/plexin*	Missense variant	Dichtel-Danjoy and Félix (2004b)
*iov-3*	*mf78*	Hyperinduction 2°	EMS	**86:**27,580–27,583	4-bp deletion	g14741	*plx-1/plexin*	Premature stop	Dichtel-Danjoy and Félix (2004b)

TMP-UV, trimethylpsoralene-ultraviolet; 2°, secondary.

a The locus was defined by complementation tests in the article cited in the last column.

b The localization corresponds to the genomic position (**scaffold**: base pair). All molecular lesions in the table were identified by the mapping-by-sequencing approach, except the additional alleles *mf78* and *sy451*, which were identified by PCR and Sanger sequencing of the gene.

All molecular loci in Table 1 were identified using two or more alleles (the identification of *Oti-lin-3* was confirmed by CRISPR alleles), except for *Oti-mig-14/Wntless* and *Oti-smp-1/semaphorin*, where we had a single mutant allele. The indication that lesions in these two genes are causal relies on the fact that other genes in the same molecular pathway were found with similar mutant phenotypes, respectively: *Oti-mom-5/Wnt receptor* and *Oti-plx-1/plexin*.

### Identification of homologous genes

The predicted protein sequences of *O. tipulae* genes were obtained through the genome annotation (Besnard *et al.* 2017), now available from the Blaxter laboratory website (http://caenorhabditis.org). The sequence of their closest *C. elegans* homolog was identified using the Basic Local Alignment Search Tool Protein (BLASTP) algorithm (Gish and States 1993), conditioning for highly similar alignments (> 80% identity) and low *e*-values. Manual curation and reannotation of the *O. tipulae* gene sequences were then performed using their closest *C. elegans* homolog as a reference. We aligned the amino acid sequences of the reannotated genes with their respective *C. elegans* homologs and outgroups using the Muscle algorithm implemented in MEGA X (Kumar *et al.* 2018) with default parameters. The phylogenetic relationship between the protein sequences was inferred using the neighbor-joining method (Saitou and Nei 1987) and tested for bootstrapping with 1000 replicates.

### Nomenclature

We followed *C. elegans* nomenclature and recommendations for other nematode species in Tuli *et al.* (2018). Briefly, mutant class names had been given at the time of our screen: *iov* for induction of the vulva, *dov* for division of VPCs, and *cov* for competence and/or centering of VPCs. Once the molecular lesion has been identified, we used the name of the *C. elegans* homolog preceded by the species prefix for *Oscheius tipulae* “Oti-”; for example the *iov-1(mf86)* allele is thus renamed *Oti-lin-3(mf86)*.

### Single-molecule fluorescence *in situ* hybridization

Single-molecule FISM (smFISH) in *O. tipulae* and in *C. elegans* was performed as previously described (Barkoulas *et al.* 2016). Mixed-stage populations were used for an mRNA localization experiment, while bleach-synchronized populations at the L3 larval stage were used for mRNA quantification. Only L3-stage nematodes with a gonad longer than 300 pixels (38.66 μm) were considered for mRNA quantification. The short fluorescently labeled oligos used in this study were acquired from LGC Biosearch Technologies and were used at a concentration of 100–200 mM. A list containing the sequences of the smFISH oligonucleotides is provided in Table S3.

### Phenotypic characterization and measurements of distances between nuclei

The cell fates acquired by the *O. tipulae* VPCs were scored as previously described (Dichtel *et al.* 2001). In summary, early L4 larvae were mounted with M9 solution on 4% agar pads containing 10 mM sodium azide and analyzed under Nomarski optics. Standard criteria were used to infer cell fates based on the topology and number of cells at different stages. The 1° fate is defined as three rounds of division with a final transverse division of each granddaughter (“TTTT”) and an attachment of the central cells to the anchor cell. Unlike in *C. elegans*, the cells with 2° and 3° fates undergo two rounds of division; as in *C. elegans* (Katz *et al.* 1995), the 2° fate is characterized by the absence of cell fusion to the epidermal syncytium and the detachment from the cuticle (“UUUU” for undivided), whereas the 3° fate is characterized by cell fusion to the epidermal syncytium, which stays attached to the cuticle (“ssss” for syncytial). The attachment to the anchor cell and to the cuticle for the 1° and 3° fates, respectively, are important features of cell fate definitions, since in Dichtel *et al.* (2001) we isolated mutants with defective divisions but no cell fate alterations in this respect; we do not study these cell division mutants here but only those where cell fates appear homeotically transformed. Half fates were assigned when two daughters of the Pn.p cells acquired distinct fates after the first cell division.

Measurements of distances between the nuclei of Pn.p cells were performed on mounted larvae at three different developmental stages: L2 molt, early L3 (before the division of dorsal uterine DU cells), and mid L3 (after DU cell division and before Pn.p divisions). The distance between the center of the Pn.p and anchor cell nuclei was measured in pixels using a Photometrics CoolSNAP ES camera and Nikon (Garden City, NY) NIS-Elements software (version 3.0.1). To avoid measurement errors due to the animal curvature, the distance between each Pn.p cell (except P6.p) and the anchor cell was calculated via a Pythagorean formula. For example, the distance between P4.p and the anchor cell is equal to:

$$\sqrt{{\left(P6.p_{AC}\right)}^{2}+{\left(P5.p_{P4.p}+P6.p_{P5.p}\right)}^{2}}$$

where$\;P6.p_{AC}$ is the distance between P6.p and the anchor cell,$\;P5.p_{P4.p}$ is the distance between P5.p and P4.p, and$\;\;P6.p_{P5.p}$ is the distance between P6.p and P5.p. Nonnormalized measurements can be found in Table S4.

### Genome editing

We followed the CRISPR-Cas9 target design in Paix *et al.* (2015). We targeted the following sequence at the *O. tipulae lin-3 cis*-regulatory region 5′-cCACCTGcatgtcctttttgcgc-3′ (E-box site in uppercase, within an underlined NGGNGG protospacer adjacent motif in the negative strand). The *mf113* allele was produced with the synthetic Oti_lin-3_A-2 -GCGCAAAAAGGACAUGCAGG- crRNA (CRISPR RNA) manufactured by Dharmacon (GE Healthcare), while *mf114* was produced with the same crRNA sequence synthesized by IDT (Integrated DNA Technologies, Inc.). Each crRNA was mixed with tracrRNA (trans-activating CRISPR RNA) (Paix *et al.* 2015) at an equimolar concentration of 200 μmol/μl. The tracrRNA:crRNA mix was incubated in a thermal ramp between 95 and 25°, decreasing by 5° every 2 min, and then mixed with purified CRISPR-Cas9 protein in HEPES buffer (pH 7.4), reaching a final concentration of 30 μM of the tracrRNA:crRNA duplex and ∼18 μM of purified protein. The final mix was incubated for 15 min at 37° and then injected into the gonad of *O. tipulae* gravid adults. The F1 progeny of the injected nematodes were placed into new plates and, after letting them lay eggs for 1 day, screened for deletions by PCR with the mf86-EboxA-F and mf86-R primers. Heterozygous F1 animals were identified by band-size separation on 3% agarose gels, and homozygous F2 mutants were easily spotted by their bag phenotype. Only a single mutation per injection session (> 10 P0s and > 200 F1s) was obtained.

To obtain *C. elegans plx-1* null mutants, hermaphrodites bearing the *plx-1(ev724)* allele were injected as above but with a crRNA sequence (5′-GGAATGCACTCAGAATGGTG-3′) that targeted a *plx-1* coding sequence located upstream of the *ev724* deletion in the second exon downstream of the second ATG, and a crRNA that targeted *dpy-10* and a short DNA oligo that introduces the *dpy-10(cn64)* as a co-CRISPR marker (Arribere *et al.* 2014). Roller F1s were isolated onto new plates, and screened for mutations in the *plx-1* gene other than the *ev724* deletion by PCR using the forward and reverse Cel-plx-1_111- primers.

### Immunofluorescence staining

Bleach-synchronized larvae and mixed-stage populations were fixed, and permeabilized for immunostaining using previously described methods (Louvet-Vallée *et al.* 2003; Kolotuev and Podbilewicz 2004, 2008). In brief, OP50-grown populations were washed three times in distilled water and placed onto poly-l-lysine-coated (P0425-72EA; Sigma [Sigma Chemical], St. Louis, MO) slides prior to freeze-cracking. Worms with an open cuticle were incubated in antibody buffer with the mouse MH27 antibody against the epithelial cell adherent junctions (Francis and Waterston 1991). This antibody was obtained from the Developmental Studies Hybridoma Bank and used at a concentration of 1 mg/ml. As secondary antibody, we used the goat anti-mouse antibody from Abcam labeled fluorescently with Alexa Fluor 488 (reference #ab150113). The slides containing immunofluorescently labeled worms were mounted with GLOX buffer (Ji and van Oudenaarden 2012) containing DAPI, covered with a cover slip, and imaged with a PIXIS camera (Princeton Instruments).

### Data availability

Supplemental Tables are available through the Figshare portal (https://doi.org/10.25386/genetics.7624559):

Table S1. List of strains used in this study.
Table S2. Sequences of DNA primers used in this study. Sequencing primers to verify by Sanger sequencing the mutations identified by the mapping-by-sequencing approach, and to identify the molecular lesions in additional alleles.
Table S3. Sequences of smFISH probes used in this study. The fluorophore coupled to each probe is noted at the end of the set name.
Table S4. smFISH quantifications, distance measurements, and vulval cell fates used in this study.

Code for mutant identification is available at https://github.com/fabfabBesnard/Andalusian_Mapping. Sequencing reads used for mapping-by-sequencing can be find at the Sequence Read Archive portal under the bioproject number PRJNA514975.

## Results

### The sole hypoinduction mutation is due to a *cis*-regulatory change in *Oti-lin-3*

**Figure 2:**
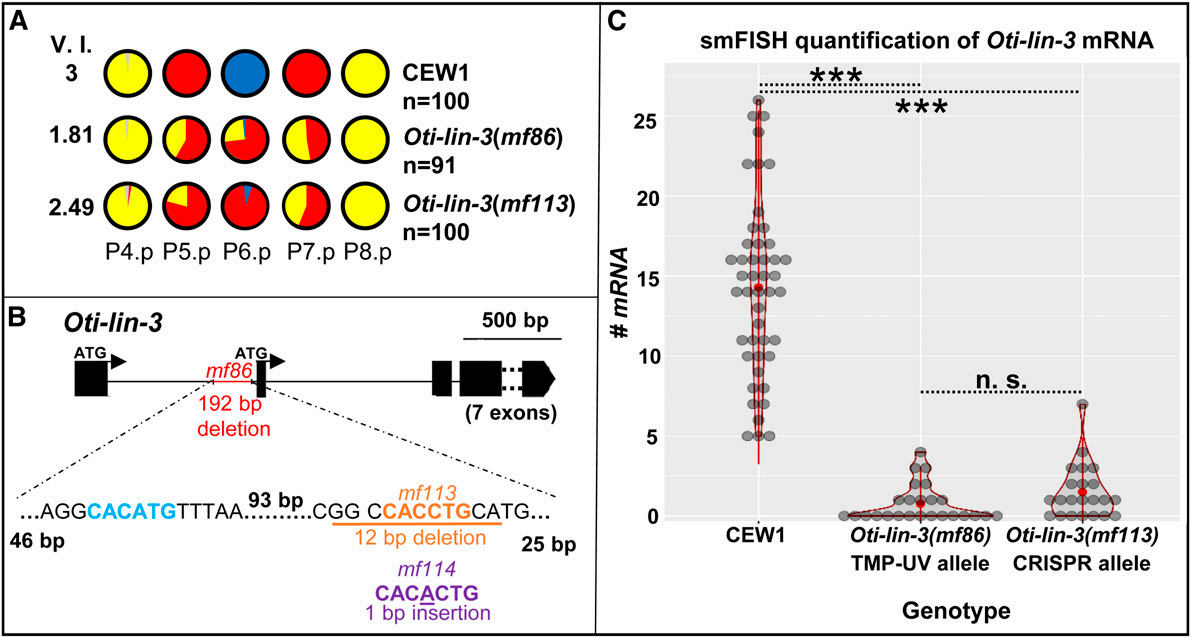
*Cis*-regulatory lesions in *Oti-lin-3*/EGF cause hypoinduction of primary (1°) and secondary (2°) vulval cell fates. (A) P(4–8) cell fates in the wild-type CEW1 *O. tipulae* reference strain and mutants for *lin-3/*EGF. The pie diagrams represent the percentage of cell fates over individuals. Yellow, red, and blue are for the tertiary (3°), 2°, and 1° fates, respectively. Gray denotes an undivided cell fused to the hypodermis. The Vulva Index (V.I.) is calculated as the average number of cells acquiring a vulval cell fate in a set of animals. The quantifications of *Oti-lin-3(mf86)* are from Dichtel-Danjoy and Félix (2004b). (B) Position of the deletions in the trimethylpsoralene-ultraviolet (TMP-UV) and clustered regularly interspaced short palindromic repeat (CRISPR) alleles. As in *C. elegans*, the *lin-3* gene of *O. tipulae* is predicted to have two alternative ATGs, with the anchor cell *cis*-regulatory element upstream of the second ATG. However, unlike *C. elegans*, *O. tipulae* has a single E-box “CACCTG” binding site and no nuclear hormone receptor (NHR) binding site. Note that the seven exons following the second ATG were excluded from the diagram. (C) Distributions of *Oti-lin-3* mRNA number in the anchor cell of wild-type CEW1 and *lin-3* *cis*-regulatory mutants, as quantified by single-molecule FISH. n.s., not significant. ***: p<0.001.

Our prior mutagenesis screens had yielded a single mutant with an Induction Vulvaless phenotype, *i.e.*, the 1° and 2° fates are transformed to a 3° fate (two rounds of division and fusion to the hyp7 syncytium, represented in yellow in the figures) but rarely to a noncompetent state (fusion to hyp7 without division, prior to the L3 stage, represented in gray in the figures) (Dichtel-Danjoy and Félix 2004b). This allele, *iov-1(mf86)*, was obtained after TMP-UV (trimethylpsoralene-ultraviolet) mutagenesis. The mapping-by-sequencing approach identified a 191-bp deletion upstream of the coding sequence (second ATG) of *O. tipulae lin-3* (*Oti-lin-3*) (Figure 2B). We hypothesized that this deletion may cause a reduced level of expression in *Oti-lin-3* and thus performed smFISH experiments to quantify *Oti-lin-3* mRNA number in the anchor cell (Raj *et al.* 2008; Barkoulas *et al.* 2013, 2016). Indeed, the *Oti-lin-3* mRNA level in the anchor cell was much decreased in animals bearing the *Oti-lin-3(mf86)* deletion compared to animals of the CEW1 reference strain (Figure 2C, Kolmogorov–Smirnov test, *P* < 10^−14^), while *Oti-lin-3* expression elsewhere in the pharynx and in the germline was retained (Figure S7B), as in the *C. elegans cis*-regulatory mutant *lin-3(e1417)* (Saffer *et al.* 2011). The deleted region in *Oti-lin-3(mf86)* contains an E-box motif known to be conserved in *Caenorhabditis* species (Barkoulas *et al.* 2016), as well as a second less-characteristic putative E-box motif (Figure 2B and Figure S7A).

To test whether the conserved E-box motif was required for the expression of *Oti-lin-3* and also confirm that the 191-bp deletion was causal for the vulva phenotype, we performed a CRISPR/Cas9 experiment specifically targeting this site. We obtained two new mutations, a smaller 12-bp deletion (*mf113*) and a 1-bp insertion in the E-box (*mf114*). Both showed a strong decrease in the level of induction, confirming that the lesion in the *Oti-lin-3* gene is causal for the phenotype (Figure 2A and Table S4). Further smFISH experiments in the *Oti-lin-3*(*mf113*) mutant revealed a similar level of mRNAs as in the *Oti-lin-3(mf86)* mutant that was different from that of the *O. tipulae* CEW1 reference strain (Kolmogorov–Smirnov test, *P* = 0.94 and *P* < 10^−9^, respectively, Figure S7B). We conclude that the conserved E-box site is required in *O. tipulae* for *lin-3* expression, and that LIN-3 secreted from the anchor cell is necessary for the induction of both 2° and 1° fates.

### The Wnt pathway plays a role in vulval precursor competence/induction and fate pattern centering

**Figure 3:**
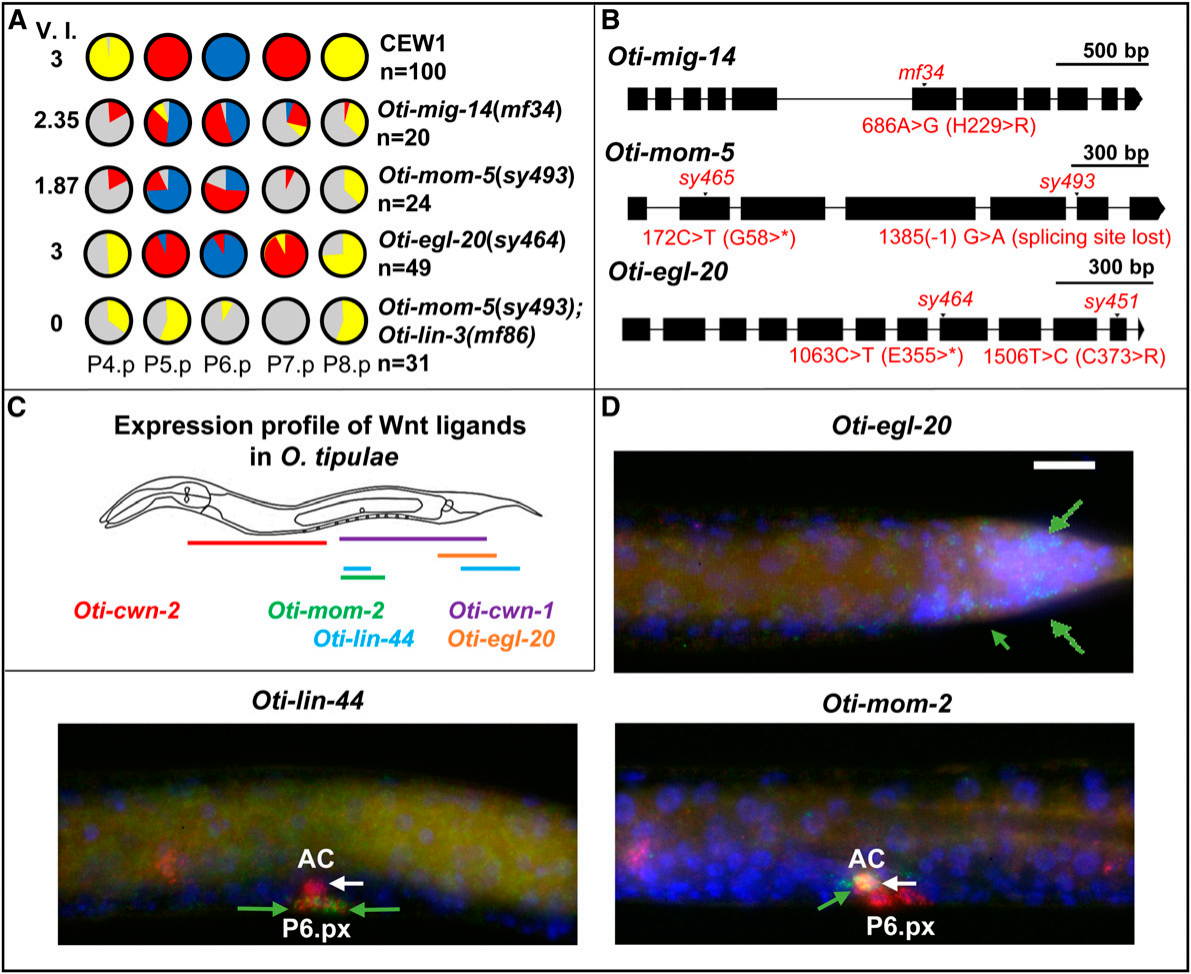
*O. tipulae* mutants in Wnt signaling display defects in competence and centering of the 1° fate on P5.p. (A) Pie diagrams representing the percentage of P(4–8).p cells acquiring one of the four possible cell fates (blue, red, yellow, and gray for the 1°, 2°, 3°, or 4° fates, respectively) for animals of different genotypes. The V.I. is calculated as the average number of cells acquiring a vulval cell fate in a set of animals. The quantifications in *Oti-mig-14(mf34)* and *Oti-mom-5(sy493*) animals are from Louvet-Vallée *et al.* (2003), and that of *Oti-egl-20(sy464)* from Dichtel *et al.* (2001). (B) Position of different mutations in genes encoding Wnt pathway components. A star designates a stop codon. (C) Diagram of Wnt ligand expression profiles in *O. tipulae* at mid-L3 stage. smFISH images of *Oti-cwn-1* and *Oti-cwn-2* are found in Figure S3. (D) smFISH images of *Oti-egl-20*, *Oti-lin-44*, and *Oti-mom-2* Wnt ligands after P6.p division at the L3 stage. mRNAs are visible as green dots. The animals were also labeled with DAPI (in blue, labeling nuclei) and fluorophore probes for *Oti-lag-2/*Δ (in red, labeling the anchor cell, P6.p descendants, and distal tip cells outside the field of view). *Oti-egl-20* is visible only in the posterior part of the animal (green arrows). *Oti-mom-2* mRNAs (green arrow) are found in the anchor cell (white arrow), while *Oti-lin-44* mRNAs (green arrows) appear in P6.p daughters (as well as sex myoblast precursors outside the focal plane). All the images are set to the same scale. The size of the white bar is 10 μm. Anterior is to the left in all images and the ventral side is down. 1°, primary; 2°, secondary; 3°, tertiary; AC, anchor cell; smFISH, small-molecule FISH; V.I., Vulva Index.

A large class of mutants in our screen displayed a lower number of competent Pn.p cells (transformation to quaternary/gray fate) and a displacement of the 1° fate from P6.p to P5.p. In *C. elegans*, this phenotype has not been seen at this high level of penetrance. The mapping-by-sequencing approach had already identified one locus in this class as *Oti-mig-13* (Besnard *et al.* 2017). In this class, we further identified mutations in two Wnt pathway components:

1. A Wnt receptor gene, *Oti-mom-5* (supported by two alleles, including an early stop before the transmembrane domain) (Figure 3B). Relationships among Wnt receptor paralogs in the different species are shown in Figure S4. Curiously, the *Oti-mom-5* putative null allele, *sy465*, is viable in *O. tipulae*, while an early stop before the transmembrane domain such as *mom-5(ne12)* is embryonic lethal in *C. elegan*s (embryonic mesoderm *vs.* endoderm specification; Rocheleau *et al.* 1997). We cannot rule out that another mutation suppresses the lethality in the *O. tipulae* strain.

2. A Wnt-processing protein, *Oti-mig-14* (homolog of *Drosophila* Wntless) (Bänziger *et al.* 2006; Yang *et al.* 2008). The *mf34* mutant displays an amino acid substitution in the *Oti-mig-14* gene and is thus likely a hypomorph that may negatively affect the activity of all Wnts. Although this gene identification relies on a single available allele at the *cov-5* locus and is thus less reliable than the above, this lesion makes *Oti-mig-14/Wntless* a very likely candidate for the causal mutation for this phenotype, since both *cov-4/Wnt receptor* and *cov-5* loci show similar mutant phenotypes (Louvet-Vallée *et al.* 2003).

We had somewhat arbitrarily distinguished classes of vulval mutations that affect competence and centering (*cov* mutants) from those that affect division of VPCs (*dov* mutants) (Dichtel *et al.* 2001). Among the latter class, we found that the *dov-4* locus encodes a Wnt-type ligand, *Oti-egl-20* (supported by two alleles, including a premature stop). The *Oti-egl-20* mutation results in a lower competence and division frequency of P4.p and P8.p, but hardly affects P(5–7).p. Centering of the 1° fate on P5.p only occurs at low penetrance. Overall, the *Oti-egl-20* phenotype is similar to that of *Oti-mig-14/cov-5* or *Oti-mom-5*, albeit much weaker, suggesting the involvement of other Wnt family ligands.

The *O. tipulae* genome contains five genes coding for Wnt signaling molecules, which we found to be 1:1 orthologs to the five Wnt genes in *C. elegans* (Figure S4). By smFISH, the expression pattern of each of these five genes was found to be quite similar in L1–L3 larvae to that of each ortholog in *C. elegans*, as determined in Song *et al.* (2010) and Harterink *et al.* (2011). Specifically, *Oti-egl-20* is expressed in the posterior region of the animal from the L1 stage (Figure 3D). *Oti-cwn-1* is also expressed quite posteriorly (Figure S3). *Oti-cwn-2* is expressed in the anterior region (Figure S3). *Oti-mom-2* is expressed in the anchor cell from the L3 stage (Figure 3D). *Oti-lin-44* is expressed in the tail region and in the L3 stage in P6.p daughters (Figure 3, Figure S3, and Figure S6). Similar to *cwn-1* in *C. elegans* (Harterink *et al.* 2011; Minor *et al.* 2013), we found that *Oti-lin-44* is additionally expressed in the sex myoblast precursors that are located to the left and right of the anchor cell in the L3 stage (Figure S3). As the sex myoblast expression of *Oti-lin-44* differed from the reported uterus/anchor cell pattern in *C. elegans* using lacZ staining or fluorescent reporters (Inoue *et al.* 2004), we localized *lin-44* by smFISH in *C. elegans* and saw similar expression in the sex myoblasts (identified by labeling with *hlh-8*::*GFP* (Harfe *et al.* 1998) and P6.px, and none in the uterus and anchor cell (Figure S6). In conclusion, the larval expression patterns of the five Wnt genes were thus similar in *O. tipulae* and *C. elegans*.

From the *Oti-egl-20* expression pattern and mutant phenotype, the EGL-20 protein is produced from the posterior of the animal and promotes Pn.p cell competence as far as P4.p. P3.p is not competent and does not divide in *O. tipulae* (Félix and Sternberg 1997; Delattre and Félix 2001), and is thus not affected by Wnt pathway mutations, whereas it is highly sensitive to Wnt pathway modulation in *C. elegans* (Pénigault and Félix 2011b). However, the difference in phenotype severity between *Oti-mig-14* or *Oti-mom-5* mutants on one hand, and *Oti-egl-20* (including the *sy464* allele with a stop codon) on the other hand, suggests that other Wnt signals, perhaps mostly CWN-1 from the posterior as in *C. elegans* (Gleason *et al.* 2006), may act jointly to promote Pn.p competence.

Overall, the major differences between *C. elegans* and *O. tipulae* for this class of mutants are: (1) Wnt pathway mutations were not found in the first vulva mutant screens in *C. elegans*; (2) the miscentering of the 1° fate on P5.p is much more penetrant in *O. tipulae* than in *C. elegans* (Figure 3, see *Discussion*); and (3) *egl-20/Wnt* mutations lead to a low division frequency of P8.p in *O. tipulae* compared to *C. elegans*, and a comparable or even weaker effect on P4.p: *Oti-egl-20(sy464)* and *Cel-egl-20(n585)* animals show 30 and 1% loss of division of P8.p, compared to 22 and 54% loss of division of P4.p, respectively (Dichtel *et al.* 2001; Myers and Greenwald 2007). Note that the *Oti-egl-20(sy464)* allele is a nonsense mutation in the Wnt domain while *Cel-egl-20(n585)* is a missense mutation, which may alternatively explain differential effects.

### The hyperinduced mutations affect plexin and semaphorin genes

**Figure 4:**
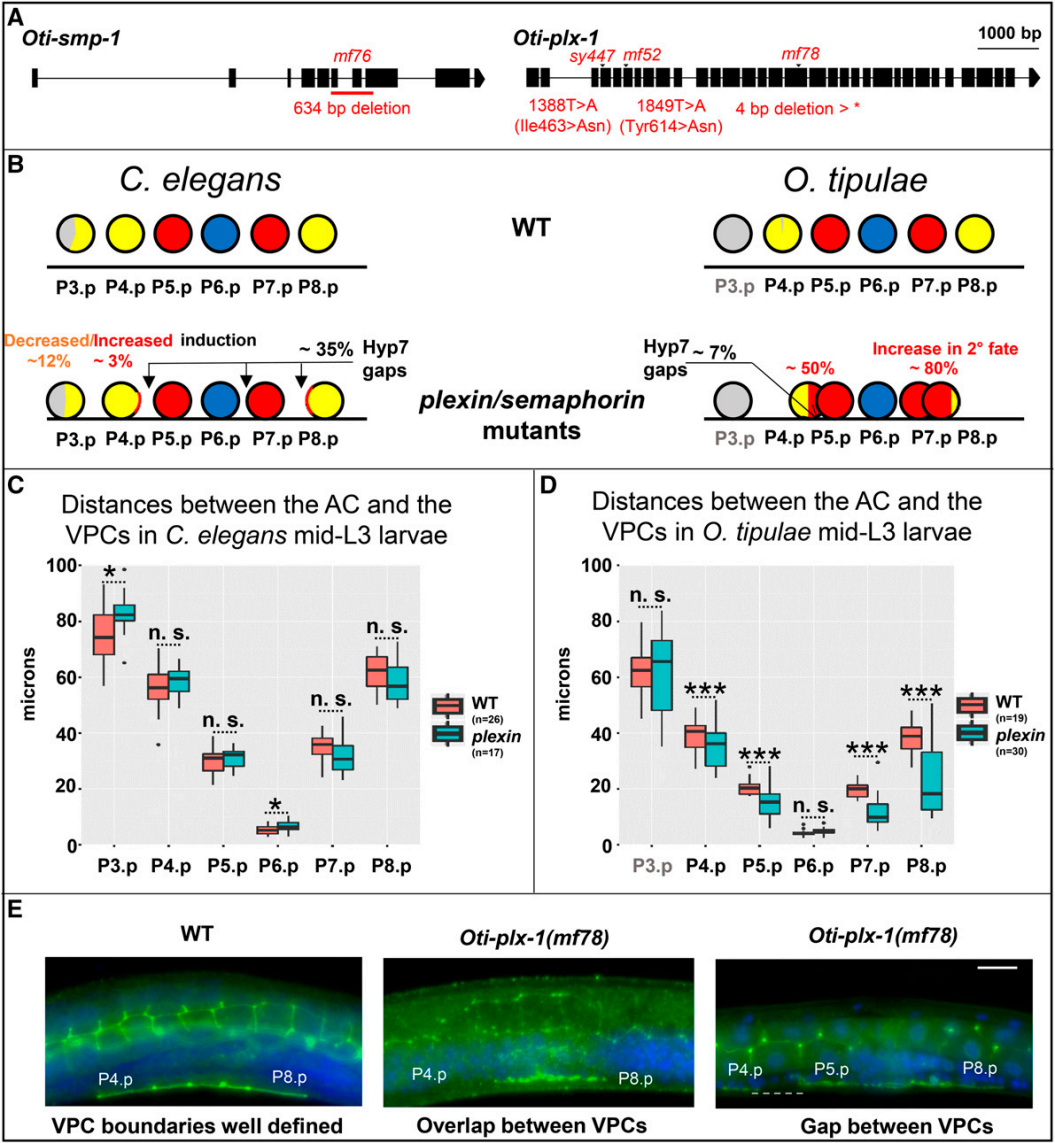
*O. tipulae* plexin/semaphorin mutants present defects in vulval induction and closer VPC cells. (A) Gene models of *Oti-smp-1* and *Oti-plx-1* with their respective mutations in *O. tipulae*. (B) Schematic depiction of the phenotypic effects of plexin/semaphorin mutants in *C. elegans* and *O. tipulae* on the induction and localization of Pn.p cells. Quantifications can be found in Table S4. In *C. elegans*, the percentages shown are the highest found along alleles, namely those in *plx-1(ev724)* mutants. Arrows show the most common localization of intercellular spaces (gaps) between VPCs. Each VPC diagram (circle) is colored according to the frequency of its acquired fate (yellow, red, and blue for the 3°, 2°, and 1° fates, respectively, and gray for undivided). Data from Dichtel-Danjoy and Félix (2004b). (C) Normalized distances between the AC and the VPCs in *C. elegans* WT and *plx-1(ev724)* animals at the mid-L3 stage after DU division. Only the distances between the AC and P3.p, and P6.p, are significantly larger in *plx-1* mutants compared to WT (Wilcoxon rank sum test, *P* < 0.05). (D) Normalized distances between the AC and the VPCs in *O. tipulae* WT and *Oti-plx-1(mf78)* animals at the mid-L3 stage after DU division. Distances between each of P(4–8).p and the AC, except for P6.p, are all significantly smaller in *Oti-plx-1(mf78)* mutants relative to WT (Wilcoxon rank sum test, with *P*-values < 10^−4^). (E) Immunostaining of cell junctions with MH27 antibody (in green), with DAPI staining in blue. The central panel shows overlapping VPCs, while the right panel shows a rare instance of a gap (dotted line) in *Oti-plx-1(mf78)* animals. All the images are set to the same scale. Bar, 10 μm. Anterior is to the left in all images, and the ventral side is down. AC, anchor cell; n.s., not significant; VPC, vulval precursor cell; WT, wild-type; *: p<0.05; ***: p<0.001.

Much more unexpected is the identification of the loci resulting in the strongest vulva hyperinduction phenotypes. We had identified two loci, *iov-2* and *iov-3*, with similar hyperinduction defects: by cell lineage and attachment criteria, P4.p and P8.p were transformed to a 2° fate at an intermediate penetrance of ∼50% for P4.p and 80% for P8.p in the strongest, TMP-UV alleles (Dichtel-Danjoy and Félix 2004b). The *iov-3* locus turned out to correspond to the *Oti-plx-1* gene, coding for a plexin (one small out-of-frame deletion as the TMP-UV allele *mf78* and two missense alleles). The *iov-2(mf76)* mutant shows a 634-bp deletion in the *Oti-smp-1* gene, coding for a semaphorin-type ligand of plexin (Figure 4A). Although this latter gene’s identification relies on a single allele at the *iov-2* locus, since both the *iov-2* and *iov-3* loci show similar hyperinduced mutant phenotypes (Dichtel-Danjoy and Félix 2004b), this lesion makes *Oti-smp-1* a very likely candidate for the causal mutation for this phenotype. This implicates a new intercellular signaling pathway in vulval cell fate patterning and induction.

The plexin-semaphorin pathway is well known for contact-dependent growth inhibition between neurons, acting in many organisms (Kolodkin *et al.* 1992; Luo *et al.* 1993; Winberg *et al.* 1998). In *C. elegans*, mutations in *smp-2/mab-20*, *smp-1*, and *plx-1* (Roy *et al.* 2000; Fujii *et al.* 2002; Ginzburg *et al.* 2002; Dalpé *et al.* 2004; Pickett *et al.* 2007; Nukazuka *et al.* 2008) were first found and most studied for their effect on the displacement of sensory organs (rays) in the male tail. Their impact on vulval formation concerns late morphogenesis events in formation of the vulval rings, events that take place after the three rounds of Pn.p divisions (Dalpé *et al.* 2005; Liu *et al.* 2005; Pellegrino *et al.* 2011). When examined, their effect on vulval induction is very weak (Liu *et al.* 2005) and mostly in the direction of hypoinduction of the 2° fate, as shown in Figure 4B and Table S4 for *plx-1* mutations. The existing *plx-1* alleles may not be null, as *nc36* and *nc37* only affect some long forms from an upstream transcriptional site, and *ev724* may lead to a truncated protein with some activity (Dalpé *et al.* 2004). Therefore, by CRISPR and nonhomologous end-joining repair in the *plx-1(ev724)* background, we generated two new alleles (*mf159* and *mf160*) corresponding to a second molecular lesion (a 26-bp deletion and an 11-bp insertion, respectively) upstream o*f ev724* in an exon corresponding to the semaphorin-binding domain. The *plx-1(mf159ev724)* and *plx-1(mf160ev724)* combinations are predicted to code only for incomplete and nonfunctional forms of PLX-1, independently of the transcriptional start site; we can conclude that these animals are true molecularly null mutants for *plx-1*. Defects in vulval induction in these animals were not stronger than those seen in *plx-1(ev724)* alone, and in this case did not include any hyperinduction (*n* = 76 and 72 for *mf159ev724* and *mf160ev724*, respectively; Table S4). To rule out that the difference in vulval phenotypes between the two species is due to a difference in paralog usage, we quantified the vulval induction in animals bearing mutations for *plx-2*, the paralog of *plx-1*; we did not see any vulval defect in the predicted *plx-2(ev773*) null mutants (*n* = 57; Table S4) (Ikegami *et al.* 2004) nor in *plx-2(ev773)*; *plx-1(ev724)* double mutants (*n* = 47; Table S4). These experiments clearly demonstrated that alterations of the plexin-semaphorin pathways in *C. elegans* do not result in vulval hyperinduction defects that are as strong as those in *O. tipulae*.

The hyperinduction of P4.p and P8.p in the *O. tipulae iov-2/smp-1* and *iov-3/plx-1* mutants is a transformation of 3°–2° fate. The ectopically induced cells never adopt a 1° fate; they join the main vulval invagination, and therefore the adult phenotype is a protruding vulva and not additional bumps on the cuticle, as in the *C. elegans* Multivulva mutants. This contrasts with the *C. elegans* hyperinduced mutants, which correspond to an excess of Ras pathway signaling, leading to ectopic 1° and 2° fates.

To understand why plexin and semaphorin mutations cause vulval hyperinduction in *O. tipulae*, we measured cell position at the time of induction, before the formation of the vulval invagination, using nuclear position as a proxy (Grimbert *et al.* 2016). As in *C. elegans* plexin and semaphorin mutants, we observed that the VPCs do not form an anteroposterior row as in wild-type animals (Dalpé *et al.* 2005; Liu *et al.* 2005; Pellegrino *et al.* 2011), but instead either overlap left and right of each other, or sometimes show a lack of a junction and a gap between successive cells (Figure 4, B–E). In contrast to *C. elegans plx-1(ev724)* mutants, gaps are rare in *O. tipulae plx-1(mf78)* mutants and do not often involve the three central cells. Instead, left–right overlaps occur between P4.p and P5.p, and between P7.p and P8.p. As these overlaps could alter the distance between the anchor cell and the Pn.p cells, we measured these distances and found that they were shorter in the *O. tipulae plx-1(mf78)* mutant, but not in the *C. elegans* counterpart *plx-1(ev724)* (Figure 4, C and D) (both alleles are deletion alleles, creating an early stop codon before the plexin domain and subjected to nonsense-mediated degradation, and thus putatively comparable null alleles). As a consequence, the VPCs tend to be closer to the anchor cell in *O. tipulae*, likely explaining the excess of 2° fate induction in the first induction wave.

In summary, the identification of these four different mutations points to the plexin-semaphorin signaling pathway being important for the correct induction of the VPCs, due to its effect on VPC positioning.

## Discussion

### The unsurprising single Vulvaless mutation in *O. tipulae*

In the initial *C. elegans* screens for vulval induction defects, most Vulvaless mutations corresponding to induction defects affected the genes *lin-2*, *lin-7*, or *lin-10* (Horvitz and Sulston 1980; Ferguson and Horvitz 1985; Ferguson *et al.* 1987). Only rare tissue-specific reduction-of-function alleles were recovered in *lin-3* and *let-23*, coding for EGF and the EGF receptor, respectively. Downstream factors in the EGFR-Ras/MAP kinase cascade were only subsequently obtained by suppressor or enhancer screens (Sternberg and Han 1998).

For *lin-3*, the first *C. elegan*s allele to be isolated, *e1417*, turned out to be a base substitution affecting a *cis*-regulatory E-box (Hwang and Sternberg 2004). The second viable allele, *n378*, is a substitution in the signal peptide, showing high tissue-specificity for reasons still unknown (Liu *et al.* 1999). Further *lin-3* alleles were obtained in noncomplementation screens or screens for lethal mutants (Ferguson and Horvitz 1985; Liu *et al.* 1999). In summary, besides the *lin-2/lin-7/lin-10* genes, a main target for a Vulvaless mutation appeared to be the *cis*-regulatory element that activates *lin-3* expression in the anchor cell in a tissue-specific manner. Given this, the sole Vulvaless mutation we found in mutagenesis of *O. tipulae*, *iov-1(mf86)*, is a remarkably predictable hit: a deletion in a noncoding region homologous to that mutated in *Cel-lin-3(e1417)* (Barkoulas *et al.* 2016). Both screens requiring that hermaphrodites be viable and fertile, we recovered a similar (orthologous) *cis*-regulatory mutation in the anchor cell element: random mutagenesis ended up being as targeted as the CRISPR/Cas9 experiment that confirmed the importance of this E-box (Figure 2).

Concerning *lin-2*, *lin-7*, or *lin-10*, we now know that the proteins LIN-2/CASK, LIN-7/Velis, and LIN-1/Mint1 bind to the C-terminus of the LET-23/EGFR receptor and help to localize it to the basolateral membrane facing the anchor cell (Simske *et al.* 1996; Kaech *et al.* 1998). Mutations in any of these three loci have so far not been recovered in *C. briggsae* and *P. pacificus*, nor here in *O. tipulae* (where screens were extensive but not saturated), whereas they are frequent in *C. elegans* (see above). Thus, it is likely that either the loss-of-function in these genes is lethal or it does not affect the vulva. It will be interesting to delete them using reverse genetic methods such as CRISPR/Cas9-mediated genome modification.

### A surprise signaling pathway found only in *O. tipulae* vulva mutant screens

In stark contrast, the identification of the plexin-semaphorin pathway using the hyperinduced mutations in *O. tipulae* was unpredictable from results in *C. elegans*, *C. briggsae* (Seetharaman *et al.* 2010; Sharanya *et al.* 2012, 2015), and *P. pacificus* (Jungblut and Sommer 1998, 2001; Schlager *et al.* 2006; Tian *et al.* 2008). In the case of *C. elegans*, the vulval fate specification errors in plexin/semaphorin mutants are indeed rare and occur at low penetrance, and in directions of both excess and loss of induction. Instead, in *O. tipulae*, the specification of P4.p or P8.p as a 2° fate is quite penetrant and only excess of induction is observed (Figure 4B and Table S4). The cell positioning defects in the *O. tipulae* plexin/semaphorin mutants explain that the hyperinduction of vulval fates is gonad-dependent (Dichtel-Danjoy and Félix 2004b). In contrast to this gonad-dependence, *C. elegans* hyperinduced mutants, such as *lin-1*, *lin-13*, *lin-15*, *lin-31*, and *lin-34(d)*, retain some vulval induction upon anchor cell ablation (or in *lin-3* double mutants) (Ferguson *et al.* 1987; Han and Sternberg 1990).

What explains the difference between *C. elegans* and *O. tipulae* in the effect of mutations in the plexin-semaphorin pathway? In both species, semaphorin and plexin appear to act via contact inhibition of the VPCs while they grow and contact each other (Liu *et al.* 2005) (Figure 4). We propose that two phenomena are involved in the fate specification difference that are not mutually exclusive. First, the VPCs are, on average, closer to the anchor cell in the early L3 stage in *Oti-plx-1* mutants compared to the corresponding *C. elegans plx-1* mutants (Figure 4, C and D; Table 2); this likely increases the exposure of P4.p and P8.p to Oti-LIN-3 from the anchor cell, hence the 2° fate. The smaller body size of *O. tipulae* may also play a role. Second, the 2° fate is in part induced in *C. elegans* by direct contact between P6.p and other VPCs through transmembrane Delta ligands. In *O. tipulae*, due to differences in the fate patterning mechanism, we have no evidence of lateral signaling, whereby the 1°-fated cell induces the 2° fate in its neighbors, nor of Notch pathway involvement, except maybe later through *Oti-delta* expression in P6.p daughters; indeed, P5.p, P6.p, and P7.p do not appear to differ from each other before their division, although this may be due to a lack of adequate markers (Félix and Sternberg 1997). Signaling from the anchor cell at a distance is thus potentially stronger in *O. tipulae* than in *C. elegans*.

**Table 2:** Quantification of large interspaces (gaps) between VPCs in *C. elegans* and *O. tipulae* plexin mutants, as determined by MH27 staining

Species	Strain	Phenotype	Number of animals
*C. elegans*	N2	WT	> 50
*C. elegans*	ST54: *plx-1(nc37)*	WT	37
		Gap P7.p–P8.p	13
		Gap P6.p–P7.p	6
		Gaps P4.p–P5.p and P6.p–P7.p	1
*O. tipulae*	CEW1	WT	> 30
*O. tipulae*	JU108: *Oti-plx-1(mf78)*	WT	35
		Gap P4.p–P5.p	2
		Gaps P4.p–P5.p and P7.p–P8.p	1

“Gap P7.p–P8.p” refers to a gap between the P7.p and P8.p cells. WT, wild-type.

In *C. elegans*, VPCs are attracted toward the anchor cell in response to LIN-3 signaling, thus creating positive feedback whereby the most induced cell moves closest to the anchor cell (Grimbert *et al.* 2016). The same feedback may be at stake for the 2° cells, but we never observed an excess of 1°-fated cells in *O. tipulae*. This correlates with the fact that we do not observe other VPCs overlapping with P6.p nor contacting the anchor cell in the plexin/semaphorin mutants. It is thus possible that lateral inhibition from P6.p to its neighbors takes place in these mutants, preventing the positioning of two VPCs below the anchor cell.

In sum, the vulval hyperinduction of plexin-semaphorin pathway mutants in *O. tipulae* is a secondary effect of VPC mispositioning, which is an unexpected finding as it does not exist in *C. elegans*. As the effect of the semaphorin pathway is on cell position, not induction *per se*, the 2° fate must be induced by another signaling pathway.

### Wnt and EGF pathways act jointly in vulval competence and induction

We find that *O. tipulae* Wnt pathway mutants affect Pn.p competence and induction (2–3° and 3° to F (fusion with hyp7 in the L2 stage) transformations, Figure 3A), and result in centering of the 1° fate on P5.p. The initial genetic screens for *C. elegans* vulva mutants did not identify the Wnt pathway. The corresponding mutants were found later by specifically screening for mutants that had a variably expressed protruding vulva phenotype (Eisenmann *et al.* 1998; Eisenmann and Kim 2000). It would be tempting to conclude that there is a difference in Wnt pathway involvement in *O. tipulae* compared to *C. elegans* vulva induction. However, we propose that the difference is subtle.

In *C. elegans*, the Wnt pathway is mostly known to maintain vulval precursor competence to receive the LIN-3 signal in the L2 and L3 stages (Eisenmann *et al.* 1998). In the absence of Wnts, the Pn.p cells adopt an F fate instead of the 3° fate (one division in the L3 stage before fusion to hyp7) (Gleason *et al.* 2006). This prevents them from being induced to a vulval fate. In other words, the Wnt signaling pathway establishes competence (F to 3° fate transformation) for the next round of signaling (EGF, which induces 1° and 2° fates). Yet the inductions by Wnt and EGF in *C. elegans* are partially intermingled. Indeed, the Wnt pathway also participates to the induction of 2° vulval fates *vs.* the 3° fate (Eisenmann *et al.* 1998; Gleason *et al.* 2002; Braendle and Félix 2008; Milloz *et al.* 2008; Seetharaman *et al.* 2010). Conversely, the LIN-3/EGF pathway participates in “competence maintenance” (F *vs.* 3°) (Myers and Greenwald 2007). Thus, both pathways appear to jointly act in *C. elegans* to promote both competence (a very first induction) and 2° vulval fate induction.

The same holds true in *O. tipulae*, with quantitative variations in mutant phenotypes. In the *Oti-lin-3(mf86)* mutant, the 1° fate is abolished while the 2° fate is reduced. The intermediate level of 2° fate may be due to some remaining *Oti-lin-3* gene expression (Figure 2C). Alternatively, another signal, such as Wnts, may participate in 2° fate induction. Accordingly, a double mutant between the EGF and Wnt pathways, *Oti-mom-5(sy493)*; *lin-3(mf86)*, abolishes induction, as in *C. elegans* (Eisenmann *et al.* 1998; Braendle and Félix 2008) (Figure 3A). Thus, we conclude that despite quantitative differences in mutant penetrance, the joint involvement of the Wnt and EGF pathways in the induction of vulval fates appears similar in *C. elegans* and *O. tipulae*.

This joint induction by Wnts and LIN-3 differs from the situation described in an outgroup nematode, *P. pacificus* (Kiontke *et al.* 2007). In this species, similar to *O. tipulae* and unlike *C. elegans*, the induction of vulval fates by the gonad occurs gradually before and after Pn.p divisions (2° then 1°) (Sigrist and Sommer 1999; Kiontke *et al.* 2007). However, in contrast to both *O. tipulae* and *C. elegans*, no EGF/Ras pathway component appears to be involved and the Wnt pathway is considered to be the only inducer from the gonad (Tian *et al.* 2008). This induction by the gonad starts before the anchor cell is born (Sigrist and Sommer 1999) and the Wnt ligand secreted by the gonad appears to be Ppa-LIN-44, based on *in situ* hybridization in the uterine precursor region (Tian *et al.* 2008) (no *Ppa-lin-44* mutant is available). Since we have now shown that the expression of *lin-44* orthologs in *O. tipulae* and *C. elegans* occurs in the sex myoblasts and not the uterine precursors (Figure 3, Figure S3, and Figure S6), it will be good to clarify the site of expression of *Ppa-lin-44* (the sex myoblasts are located on either side of the uterus, and fixation for *in situ* hybridization followed by alkaline phosphatase reaction may render the distinction between uterus and sex myoblast expression difficult). This is important, as Ppa-LIN-44 can represent the vulval induction signal as proposed only if it is expressed in the gonad precursors ablated in Sigrist and Sommer (1999). Indeed, the only other Wnt ligand expressed in the *P. pacificus* gonad is *Ppa-mom-2*, but its expression in the anchor cell appears to start much later than when the induction of 2° fates begins (Sigrist and Sommer 1999; Kiontke *et al.* 2007; Tian *et al.* 2008).

Another argument in Tian *et al.* (2008) regarding the Wnt pathway being the main vulva-inducing pathway in *P. pacificus* comes from the high penetrance of the *Ppa-bar-1/armadillo(0)* mutant compared to its *C. elegans* counterpart (Tian *et al.* 2008). However, only two β-catenins were found in *P. pacificus*, as there are no *wrm-1* or *sys-1* orthologs (Tian *et al.* 2008). The null mutation in the *armadillo* homolog gene that is called *Ppa-bar-1* was obtained by a targeted reverse genetic approach and is maternal-effect lethal, unlike in *C. elegans*. In *C. elegans*, *wrm-1*; *bar-1* double mutants were noted by Green *et al.* (2008) to be fully Vulvaless. Thus, the strong vulval phenotype of the *Ppa-bar-1* mutant could in part be explained by less *armadillo* gene redundancy in *P. pacificus* than in *C. elegans* and *O. tipulae*. Upstream of these *armadillo* paralogs, in *P. pacificus* as in *C. elegans*, the multiple Wnt ligands and receptors are partially redundant (Gleason *et al.* 2006; Tian *et al.* 2008). In *C. elegans*, triple mutants in Wnts have a strong vulval phenotype, including loss of division characteristic of the 3° fate in *C. elegans*. Note that there is no equivalent to the 3° fate in *P. pacificus*; on the anterior side, the noncompetent cells die by apoptosis, and on the posterior side, P8.p is competent early on to replace P(5–7).p then fuses to hyp7 without division after the onset of vulval induction, which occurs earlier than in *C. elegans* compared to larval molts (Sommer 1997; Sigrist and Sommer 1999; Jungblut and Sommer 2000).

In sum, there is so far no indication that the induction by LIN-3 seen in *O. tipulae* and *C. elegans* is present in the outgroup *P. pacificus*. Further work is needed to substantiate this result and, most interestingly, systematic phylogenetic analysis with more outgroup species is required to polarize the character.

### The Wnt pathway is required for correct centering of the vulval pattern

The clearest difference of Wnt pathway mutant phenotypes between *C. elegans* and *O. tipulae* lies in the centering of the 1° fate on P5.p, and the likely correlated higher penetrance of the F fate in P7.p. In *C. elegans*, only a small percentage of Wnt pathway mutant animals display P5.p centering, which has been shown to reflect the posterior displacement of P6.p compared to the anchor cell and higher variance in cell positions (Milloz *et al.* 2008; Grimbert *et al.* 2016). In *Oti-mom-5* animals, a strong shift in anchor cell position relative to P6.p and P5.p in the L2 stage has also been observed (Louvet-Vallée *et al.* 2003). Quantitative differences between the various phenotypes in the two species likely correspond to the extent of cell displacement.

The phenotypes of *Oti-mom-5/cov-4* and *Oti-mig-14/cov-5* mutants closely resemble that of *Oti-mig-13/cov-3*, the homolog of mammalian low-density lipoprotein receptor-related protein 12 identified by Besnard *et al.* (2017). These three loci were grouped in the same *O. tipulae* phenotypic subclass in Louvet-Vallée *et al.* (2003), corresponding to a loss of VPC competence and miscentering of the 1° fate on P5.p. In *C. elegans*, *mig-13* mutations were first identified as affecting the anterior migration of QR neuroblasts (Sym *et al.* 1999), a process that is also affected at many steps by Wnt pathway mutations including *mom-5* and a noncanonical Wnt pathway for the long-range migration (Rella *et al.* 2016). *mig-13* acts cell autonomously in the migrating QR neuroblast lineage, where its expression is under direct positive regulation by the *lin-39* Hox gene (Wang *et al.* 2013). The MIG-13 protein is located at the plasma membrane of migrating QR neuroblasts, and is required for coronin and actin cytoskeleton polarization (Wang *et al.* 2013). We previously showed that *C. elegans mig-13* mutants show a mild phenotype of VPC competence loss and miscentering of the 1° fate on P5.p (Besnard *et al.* 2017). The relationship between the Wnt pathway and MIG-13 in vulval development remains unclear, but it is tempting to speculate that, as for QR neuroblasts, they may jointly affect VPC polarity, growth, and movement via the actin cytoskeleton (Wang *et al.* 2013; Grimbert *et al.* 2016).

### Conclusions and future directions

This study reveals the two faces of genetic screens: necessity and contingency. On the side of necessity, *Cel-lin-3(e1417)* and *Oti-lin-3(mf86)* were obtained among the first mutations defective in anchor cell induction of the vulva, both reducing the function of a tissue-specific *cis*-regulatory element of the inductive signal. This convergent result exemplifies the power and near predictability of genetic screens in targeting mutations in a lethal pathway in a tissue-specific manner. On the side of contingency, the mutations causing hyperinduction of the vulva hit contrasting molecular pathways in *C. elegans* *vs.* *O. tipulae*, contingent on particular features of the genetic architecture and anatomy of each species. For example, in *C. elegans*, initial discovery of *lin-12/Notch* involvement relied on rare gain-of-function mutations (Greenwald *et al.* 1983), and the finding of the Synthetic Multivulva genetic pathway hinged on the *lin-15* operon and the chance double hit of the *lin-8*; *lin-9* mutant (Ferguson and Horvitz 1985, 1989; Clark *et al.* 1994; Huang *et al.* 1994; Saffer *et al.* 2011). In *O. tipulae*, those same pathways could also be important but less easily targeted in vulval screens (which are not yet saturated), while differences in cell positioning may explain the stronger requirement for the plexin-semaphorin signaling pathway.

**Figure 5:**
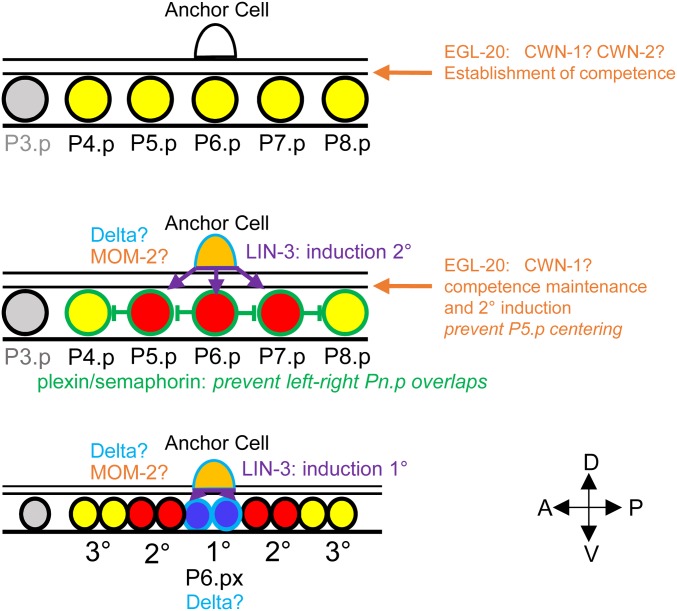
Expression of signaling molecules and vulval cell fate patterning in *O. tipulae*. The VPCs are color-coded according to their fate as in previous figures. Their boundary is color-coded according to the signaling molecules that they express (at least as mRNAs): LIN-3 in purple, Wnts in orange, plexin in green, and Delta in light blue. A question mark indicates that the effect of removing this signal is not known. Note that in addition, the sex myoblasts left and right of the AC express *lin-44/Wnt*. 1°, primary; 2°, secondary; 3°, tertiary; A, anterior; AC, anchor cell; D, dorsal; P, posterior; V, ventral; VPC, vulval precursor cell.

Figure 5 presents our current model of the vulval cell fate patterning mechanism in *O. tipulae*. Oti-LIN-3 produced by the anchor cell is important for the induction of 2° then of 1° fates. Oti-LIN-3 is thus likely the inductive signal for both steps of induction, as defined in Félix and Sternberg (1997). The 1° fate induction appears to always occur upon contact with the anchor cell, which may represent a requirement for a transmembrane ligand or simply a high concentration of the ligand. Thus, although the cell fate patterning mechanism unraveled through anchor cell ablation is derived in *C. elegans* compared to many other nematode species (Kiontke *et al.* 2007), the involvement of LIN-3 in vulval induction appears to be older.

Our findings on the effects of both the Wnt and semaphorin pathways on VPC positioning relative to the anchor cell emphasize the importance of cell positioning in vulval cell fate patterning, since gradients of signaling molecules (EGF and Wnt) are involved. The VPC positioning defect may link the Wnt and MIG-13 pathways to cell polarity, growth, movement, and the actin cytoskeleton (Wang *et al.* 2013; Grimbert *et al.* 2016).

Finally, a future direction concerns Notch pathway involvement. It may not be surprising that mutations in this pathway were not found in the screens as they are sterile or lethal in *C. elegans*. In *P. pacificus*, the Notch pathway appears relevant to intraspecific evolution (Kienle and Sommer 2013), but no mutant was found or produced by reverse genetics. However, we do not rule out their possible involvement, in light of *dsl* expression in P6.p daughters and the role of *apx-1* in intraspecific evolution in *P. pacificus* (Kienle and Sommer 2013).
